# Draft Genome Sequence of Marine-Derived Actinomycete *Nocardiopsis* sp. Strain TP-A0876, a Producer of Polyketide Pyrones

**DOI:** 10.1128/genomeA.00665-14

**Published:** 2014-07-10

**Authors:** Hisayuki Komaki, Natsuko Ichikawa, Akira Hosoyama, Nobuyuki Fujita, Yasuhiro Igarashi

**Affiliations:** a Biological Resource Center, National Institute of Technology and Evaluation (NBRC), Chiba, Japan; b NBRC, Tokyo, Japan; c Biotechnology Research Center and Department of Biotechnology, Toyama Prefectural University, Toyama, Japan

## Abstract

Here we report the draft genome sequence of *Nocardiopsis* sp. strain TP-A0876, isolated from marine sediment, which produces polyketide-derived pyrones called nocapyrones. The genome contains three polyketide synthase (PKS) gene clusters, one of which was proposed to be responsible for nocapyrone biosynthesis. This genome sequence will facilitate the study of the potential for secondary metabolism in *Nocardiopsis* strains.

## GENOME ANNOUNCEMENT

Marine actinomycetes are promising sources of bioactive compounds (1–3). In our screening for discovering new adiponectin inducers, *Nocardiopsis* sp. strain TP-A0876 was isolated from marine sediment and found to produce four pyrone compounds of polyketide origin: nocapyrones B, H, L, and R (14). To identify their biosynthetic gene cluster, we performed whole-genome shotgun sequencing of the strain. Herein we present its draft genome sequence.

*Nocardiopsis* sp. TP-A0876 was deposited to the NBRC culture collection and registered as NBRC 110039. The whole genome of *Nocardiopsis* sp. TP-A0876 was read by using a combined strategy of shotgun sequencing with GS FLX+ (Roche; 20 Mb sequences, 3.5-fold coverage) and pair-end sequencing with MiSeq (Illumina; 772 Mb, 128-fold coverage). These reads were assembled using Newbler v2.6 software and subsequently finished using GenoFinisher software (4), which led to a final assembly of 25 scaffold sequences of >500 bp each. The total size of the assembly was 5,861,114 bp, with a G+C content of 68.8%. Coding sequences were predicted by Prodigal (5), and domains related to polyketide synthase (PKS) and nonribosomal peptide synthetase (NRPS) were searched for using the SMART and PFAM domain databases. PKS and NRPS gene clusters and their domain organizations were determined manually. The genome was found to contain two type-I PKS gene clusters, one type-II PKS gene cluster, one NRPS gene cluster, and one hybrid PKS/NRPS gene cluster.

The 16S rRNA gene sequence (GenBank accession no. AB488799) of *Nocardiopsis* sp. TP-A0876 showed 100% identity to that of *Nocardiopsis alba* DSM 43377^T^. To examine the taxonomical identity of our strain with this species, the average nucleotide identity (ANI) was analyzed by JSpecies (version 1.2.1) (6), using currently available genome sequences of two *N. alba* strains, drainage-derived DSM 43377^T^ (7) and honeybee gut-derived ATCC BAA-2165 (8). The ANI values between strain TP-A0876 and the two *N. alba* strains were 98.35% to 99.04%. Based on these similarities, TP-A0876 was identified as *N. alba* (9). The two *N. alba* strains also possess gene clusters homologous to the five PKS and NRPS gene clusters found in the TP-A0876 genome.

During our genome analysis of *Nocardiopisis* sp. TP-A0876, Lin et al. reported a putative nocapyrone biosynthetic gene cluster, *ncpA-D*, in *N. alba* CR167, isolated from the venom duct of a cone snail (10). One (*orf181* to *orf178* in scaffold009) of the two type-I PKS gene clusters in *Nocardiopisis* sp. TP-A0876 is homologous to *ncpA-D*. We assume that this cluster is responsible for nocapyrone biosynthesis in strain TP-A0876. Interestingly, a variety of pyrone derivatives were isolated from marine-derived *Nocardiopsis* strains (11–14). The actual products of the remaining four PKS and NRPS gene clusters have not been isolated or characterized from our strain. The genome sequence of *Nocardiopisis* sp. TP-A0876 will serve as a valuable reference to elucidate the potential of marine-derived *Nocardiopisis* strains as promising sources for bioactive secondary metabolites.

### Nucleotide sequence accession numbers.

This whole-genome shotgun project has been deposited in DDBJ/ENA/GenBank under the accession no. BAZE00000000. The version described in this paper is the first version, BAZE01000000.
